# Optimization and Characterization of Electrodeposited Cadmium Selenide on Monocrystalline Silicon

**DOI:** 10.3390/nano12040610

**Published:** 2022-02-11

**Authors:** Walter Giurlani, Martina Vizza, Antonio Alessio Leonardi, Maria Josè Lo Faro, Alessia Irrera, Massimo Innocenti

**Affiliations:** 1 Dipartimento di Chimica, Università degli Studi di Firenze, Via della Lastruccia 3, 50019 Sesto Fiorentino, Italy; walter.giurlani@unifi.it (W.G.); martina.vizza@unifi.it (M.V.); 2 INSTM, Consorzio Interuniversitario Nazionale per la Scienza e Tecnologia dei Materiali, Via G. Giusti 9, 50121 Firenze, Italy; 3 URT LAB SENS, Beyond Nano—CNR, c/o Department of Chemical, Biological, Pharmaceutical and Environmental Sciences, University of Messina, Viale Ferdinando Stagno d’Alcontres 5, 98166 Messina, Italy; antonio.leonardi@dfa.unict.it (A.A.L.); mariajose.lofaro@dfa.unict.it (M.J.L.F.); irreraalessia@gmail.com (A.I.); 4 CNR-IPCF, Istituto per i Processi Chimico-Fisici, V.le F. Stagno D’Alcontres 37, 98158 Messina, Italy; 5 Dipartimento di Fisica ed Astronomia, Università di Catania, Via Santa Sofia 64, 95123 Catania, Italy; 6 CNR-ICCOM, Istituto di Chimica dei Composti OrganoMetallici, Via Madonna del Piano 10, 50019 Sesto Fiorentino, Italy; 7 CSGI, Center for Colloid and Surface Science, Via della Lastruccia 3, 50019 Sesto Fiorentino, Italy

**Keywords:** CdSe, cadmium, selenide, silicon, optoelectronics, thin film, electrodeposition

## Abstract

In this work, the optimal conditions for the electrodeposition of a CdSe film on n-Si were demonstrated. The structural and optical properties of the bare films and after annealing were studied. In particular, the crystallinity and photoluminescence of the samples were evaluated, and after annealing at 400 °C under a nitrogen atmosphere, a PL increase by almost an order of magnitude was observed. This paper opens the route towards the use of electrochemical deposition as a cost-effective and easy fabrication approach that can be used to integrate other interesting materials in the silicon-manufacturing processes for the realization of optoelectronic devices.

## 1. Introduction

Silicon is the leading material of contemporary technology as we know it and presumably will remain a fundamental pillar in the future as well. The use of silicon in microelectronics is currently undiscussed. However, the indirect bandgap of silicon makes the realization of Si-based devices with integrated photoemission complex, even if this is a fundamental step in the integration of photonics and microelectronics. Doped silicon itself has low photoemission and various tactics have been used to improve this aspect such as defect emission [1,2,3], fluorescent rare-earth doping [4,5,6], and quantum confinement effect [7,8,9]. In recent years, the use of porous silicon and silicon nanowires (NWs) has achieved good results in emission at room temperature [10,11,12,13]. A different approach consists of coupling other semiconductors to silicon in order to exploit their photoemission characteristics [14]. The integration of different semiconductors on the same support would allow for the production of extremely small and economical devices. In particular, nanoparticles made of II–VI semiconductors are currently being extensively studied due to their unique size-dependent properties. In this framework, CdSe nanoparticles show enhanced luminescence, increased oscillator strength and shorter response time, fostering the interest in energy and optoelectronics applications [15]. Cadmium selenide is a good candidate for these purposes as it has good optical characteristics [16,17,18], is used in solar cells [19], light-emitting diodes (LEDs) [20,21,22], laser diodes [23,24,25], photo- and electro- luminescent devices [26,27], and fluorescent sensors [28,29,30]. Additionally, CdSe versatile material can also be used in its pure nanocrystalline form [31], or in core–shell combination with other metals [32,33], or even in polymer composites to further improve its chemical-physical properties, such as CdSe/TiO2 [34] or In2Se3/CdSe nanocomposites [35] for energy applications.

The cheapest and industrially scalable method for depositing CdSe is electrodeposition, which can be conducted at ambient temperature and pressure, unlike steam techniques, while still maintaining a very fine control over the quantity and characteristics of the deposited material.

The semiconductor nature of Si makes finding the right deposition conditions a difficult task since the exchange of electrons between the electrode and the solution is severely limited compared to metal electrodes and is influenced by the lighting conditions. While the deposition of CdSe on metals is now a well-known practice in the scientific field [36,37,38,39], as well as other semiconductors (CdS [40], MoSe_2_ [41], Bi_2_Se_3_ [42]), its deposition on Si has been scarcely explored.

In a previous work, we evaluated the possibility of obtaining continuous films of CdSe on commercial n-Si (100) by electroplating at room temperature [43]. In this study, we investigated this aspect by looking for the optimal conditions for the codeposition of Cd and Se to obtain high efficiency and maintaining a nanometric thickness. Rutherford backscattering spectrometry (RBS) measurements were used to determine the deposited atomic density of the components. In addition, annealing processes were carried out to assess whether a structural rearrangement could lead to greater crystallinity, and the resulting samples were characterized by an Atomic Force Microscopy (AFM) and analysed in terms of absorption and photoluminescence (PL) showing an enhanced PL after the optimized thermal treatment.

## 2. Materials and Methods

### 2.1. Electrochemical Measurements

The codeposition solutions were prepared using ultrapure MilliQ water (18 MΩ, Merk Millipore, Burlington, MA, USA) with 0.1 mM of Na_2_SeO_3_ and 3CdSO_4_∙8H_2_O and H_2_SO_4_ 0.1 M (Sigma-Aldrich, St. Louis, MO, USA). The solutions were deaerated with nitrogen and stored under nitrogen atmosphere in sealed Pyrex jars. For the deposition, we used a PC-controlled automated deposition system [44]. The capacity of the cell was 1.88 mL. The working electrode was an n-Si 100 (P-doped with a resistivity of 1–5 Ω∙cm) with a diameter of 1 cm. Before each deposition, the electrode was cleaned following the RCA procedure [43,45]. The electrochemical depositions were carried out at room temperature in the dark to exclude the influence of light that could potentially lead to the photoexcitation of silicon. All the given potentials refer to the Ag/AgCl sat. KCl electrode.

### 2.2. Microscopic and Spectroscopic Characterization

The scanning electron microscopy (SEM) images were acquired using a S-2300 Hitachi (Tokyo, Japan) equipped with a Thermo Fisher Scientific Noran System 7 detector (Waltham, MA, USA) to perform the semiquantitative microanalysis (EDS) and analysed with Pathfinder 2.1 software (Thermo Fisher Scientific, Waltham, MA, USA). The analyses were performed with an accelerating voltage of 20 kV and the stage was tilted by 45° to emphasize the 3D shapes.

The Rutherford Backscattering Spectrometry (RBS) was carried out by using a He+ beam at an energy of 2 MeV, spectra were analysed using SIMNRA 7.03 software (Max-Planck-Institut für Plasmaphysik, Garching, Germany). After that the beam impinged onto the sample, the backscattered He^+^ ions were collected at the detection angle of 165° with respect to the beam direction. Finally, a multichannel analyser was used to measure the energy loss of the backscattered ions. The crystallinity of the deposit was characterized using a Bruker (Billerica, MA, USA) New D8 Da Vinci Diffractometer to perform X-ray Diffraction spectroscopy (XRD) with Cu K radiation, Ni filter, fast multichannel energy-discriminator detector, flat holder, and Bragg-Brentano configuration in the 20° and 60° range. DIFFRACT.EVA 5.2.0.5 Bruker (Billerica, MA, USA) software was used for the interpretation of the diffractograms. The XRD analyses were performed on the samples as prepared and after the heat treatment of 1 h at 200 °C, 1 h at 400 °C, or 4 h at 400 °C. The annealing was performed in a furnace under nitrogen flux.

The room-temperature emission of the samples was tested by photoluminescence (PL) spectroscopy using a HR800 Spectrometer (HORIBA Ltd, Kyoto, Japan) and the 476 nm line of an Ar^+^ laser as excitation focused onto the sample through a 100X (0.9 NA) objective. The room temperature emission of the samples was then analysed by a Synapse Peltier cooled CCD detector (HORIBA Ltd, Kyoto, Japan). This setup works in a backscattering configuration and the same objective was used to acquire the signal.

## 3. Results

**Figure 1:**
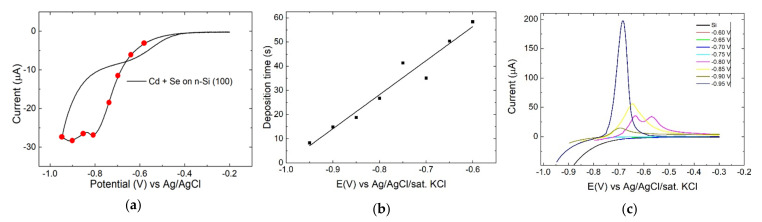
(**a**) CV of Cd^2+^ and Se (IV) solution on n-Si in sulfuric acid between −0.2 V to −0.95 V, scan rate 10 mV/s. (**b**) Dependence between applied potential and time required for a 30 mC deposition. needed for the deposition; (**c**) Linear stripping voltammetry of the samples obtained at various potentials between the deposition potential and −0.3 V, scan rate 10 mv/s.

The cyclic voltammetry (CV) of the solution containing cadmium and selenium was reported in Figure 1a. The deposition on the n-Si substrate slowly began at −0.5 V reaching a first cathodic peak at −0.8 V a second peak at −0.91 V, the deposition appears irreversible since no anodic peaks were detected. The −0.8 V peak was assigned to the codeposition of CdSe, while the peak at −0.91 V corresponds to the deposition of Cd [43,46]. We performed a charge-controlled deposition at eight different potentials to evaluate the changes in the deposit at each point. The chosen potential ranged from −0.60 V to −0.95 V every −0.05 V. The depositions were performed by depositing a charge of 30 mC independently from the applied potential. Every 1 mC, fresh solution was injected in the cell to keep the concentration on the surface of the electrode constant, the procedure was repeated 30 times. Based on the total deposited charge, the density of CdSe, and the dimension of the electrode, the deposit should roughly have had a thickness of 21.7 nm.

The time required for deposition followed a linear trend as shown in Figure 1b. To evaluate the nature of the deposits, we performed a stripping between the deposition potential and 0.5 V (Figure 1c), even an uncoated silicon substrate was measured for comparison. At potentials greater than −0.25 V, every sample, including the bare n-Si, showed an anodic current. In the range of potentials between −0.75 V and −0.50 V the samples obtained with a deposition potential lower than −0.8 V exhibited an anodic peak produced by the presence of excess Cd.

New and fresh samples were prepared for further characterizations.

**Figure 2:**
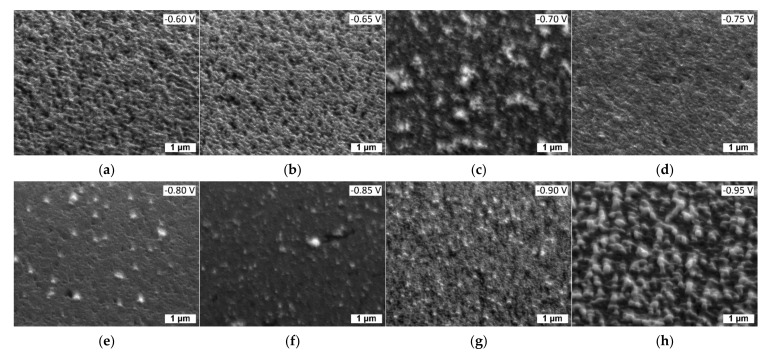
SEM images using the stage tilted of 45° of the samples of CdSe obtained ad the following potentials: (**a**) −0.60 V; (**b**) −0.65 V; (**c**) −0.70 V; (**d**) −0.75 V; (**e**) −0.80 V; (**f**) −0.85 V; (**g**) −0.90 V; (**h**) −0.95 V.

An SEM analysis was performed on each sample (Figure 2a–h). The deposition was not fully homogeneous but appeared to be quite smooth considering that to obtain an appreciable image the stage had to be tilted of 45° and the contrast was set to almost maximum. Such difficulties could be also attributed in part to the low thickness of the coatings (around 10 nm).

Samples obtained with a potential of −0.60 V (a) and −0.65 V (b) present come holes on the surface, attributable to the non-fusion of the growth nuclei, probably caused by the low deposition potential. Samples realized with a deposition potential between −0.70 V and −0.90 V are quite uniform and similar one to each other. The deposition performed at −0.95 V shows the formation of several clusters; comparing this result with the electrochemical data, we suppose their reflects excess of Cd deposited at higher overpotentials.

**Figure 3:**
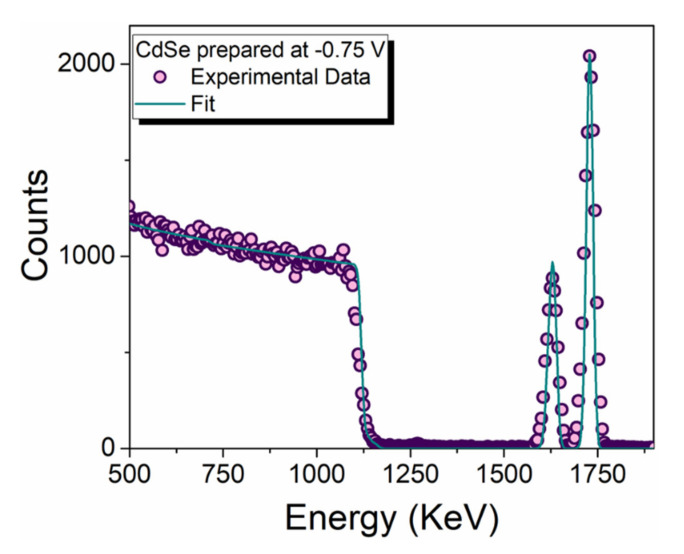
RBS analysis of the CdSe sample prepared at −0.75 V on the Si substrate.

The samples were analysed with RBS (Figure 3) to obtain the amount of Cd and Se present on the sample. RBS allows for measuring the surface atomic concentration (atm ∗ cm^−2^) of cadmium and selenium on the electrode. With the surface atomic concentration, it is possible to determine two very important characteristics of the samples: the stoichiometric ratio and the thickness. Considering the stoichiometric ratio 1:1 in CdSe the equivalent amount of cadmium selenide was deduced (Equation (1)), excluding the excess of Se or Cd.

(1) $$\mathrm{CdSe}\%=\frac{2\cdot \min\left(\left[\mathrm{Cd}\right],\;\left[\mathrm{Se}\right]\right)}{\left[\mathrm{Cd}\right]+\left[\mathrm{Se}\right]}$$

We found that the percentage of CdSe is >99% for deposition potential was lower than −0.70 V (Table 1). At a lower overpotential, we obtained an excess of selenium. Using only this information, it is not possible to distinguish if CdSe is deposited or if the elements are present in the form of Cd^0^ and Se^0^. From the electrochemical measurements, we observed that for a potential lower than −0.80 V, an excess of Cd was deposited as observed from the stripping voltammetry (Figure 1c). 

RBS is also an established technique for the determination of the thickness [47], and by knowing the surface atomic concentration and the density of the deposit, it is easy to calculate the thickness of the coating (Equation (2)):

(2) $$t_{\mathrm{CdSe}}=\frac{M\cdot \min\left(\left[Cd\right],\;\left[Se\right]\right)}{N_{A}\cdot \rho }$$

where N_A_ is the Avogadro number, ρ the density and M the molar mass.

Considering the density of the compound and the geometric area of the electrode the equivalent thickness was calculated and the efficiency (ε%) (Equation (3)) was obtained by comparing the experimental value of the sample (t_CdSe_) with the theoretical one (t_t_).

(3) $$\varepsilon \%=\frac{t_{CdSe}}{t_{t}}$$

The theoretical thickness was calculated using the Faraday law (Equation (4)) considering the amount of deposited charge (Q = 30 mC), the molar mass (M = 191.37 g/mol) and density (ρ = 5.82 g/cm^3^) of CdSe, the area of the electrode (A = 0.785 cm^2^), the number of electrons (n = 6) and the Faraday constant (F = 96,485 C/mol).

(4) $$t_{t}=\frac{Q\cdot M}{A\cdot \rho \cdot n\cdot F}=21.7\;nm$$

The deposition potential of −0.70 and −0.75 V produced the highest deposition thickness with the highest deposition efficiency. From these results, we decided to elect the −0.75 V as the best operating condition to obtain the CdSe film on n-Si. In the RBS spectrum of the −0.75 V CdSe sample is reported showing the experimental data along with the obtained fit. 

We prepare fresh samples using this potential and we performed XRD and PL on the samples as prepared and after annealing treatment.

**Figure 4:**
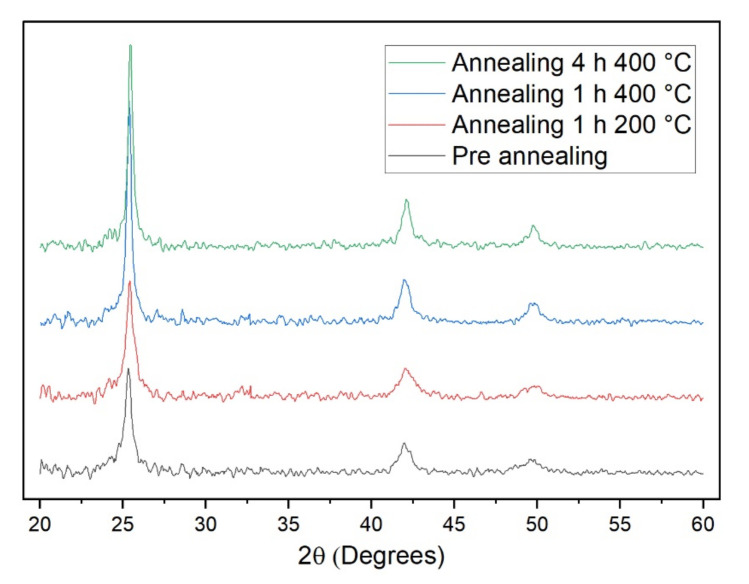
XRD analysis of CdSe samples prepared with a potential of −0.75 V (black) and then annealed under N_2_ atmosphere for 1 h at 200 °C (red), 1 h at 400 °C (blue), or 4 h at 400 °C (green).

Samples prepared using a potential of −0.75 V were annealed under N_2_ atmosphere using the following conditions: 1 h at 200 °C, 1 h at 400 °C, or 4 h at 400 °C. Then, the crystallinity of the samples was investigated with XRD (Figure 4). The as-prepared sample shows the peaks characteristic of cubic CdSe at 25.5°, 42.2, and 49.9° [48,49]. No other signal was observed, meaning that the stoichiometric ratio between the two elements was satisfied. After annealing at 200 °C, we did not observe any substantial change. In both the two diffractograms recorded on the samples annealed at 400 °C, the peaks are more intense and sharper, even if the change in intensity does not differ considerably from the pre-annealed sample. Moreover, the number of peaks and their position remains unaltered. The low intensity and the lower number of peaks compared to bulk CdSe can be assigned to the very low amount of deposited substance. These results suggest that the thermal treatment favours a rearrangement of the atoms towards a more crystalline structure, but also the deposited films were not completely amorphous. Since the peaks do not change their positions we can assert that we do not have a change in crystalline structure from cubic to hexagonal, as sometimes observed by other authors [50,51,52]. In particular, we did not observe any variation at 23.9° in correspondence of the most intense and characteristic [100] peak of the hexagonal CdSe. No substantial differences were observed between the two different annealing time at 400 °C.

**Figure 5:**
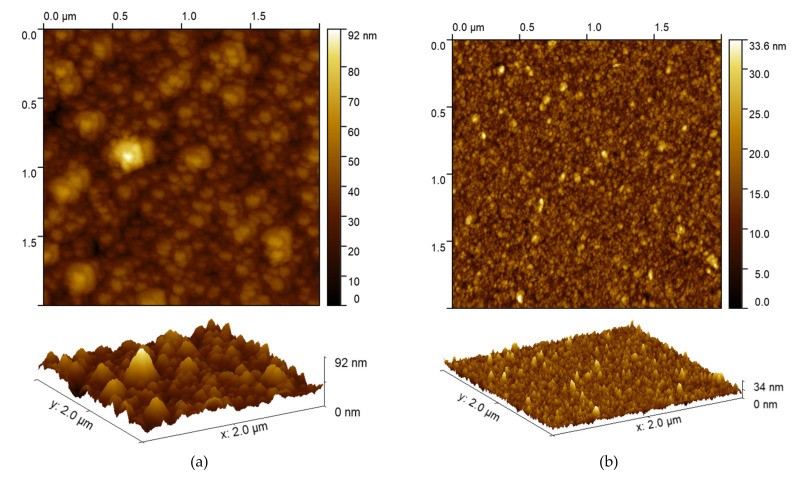
2D and 3D AFM analysis of the sample prepared at −0.75 V before (**a**) and after the 4 h annealing performed at 400 °C (**b**).

An AFM analysis was performed on the sample prepared with a deposition potential of −0.75 V, as growth was observed and after the 4 h thermal annealing performed at 400 °C (Figure 5). After the heat treatment, we observed a change in roughness, and after annealing the sample was flatter with a decreasing in roughness of almost three times while the maximum peak height dropped from 92 nm to 33.6 nm, while the RMS roughness (Sq) decreased from 9 nm to 3.5 nm. Considering that from the XRD measurements there is only a slight improvement of the crystallinity we can deduce that there is an arrangement of the matter towards a flatter condition, but the size of the grains does not change much.

**Figure 6:**
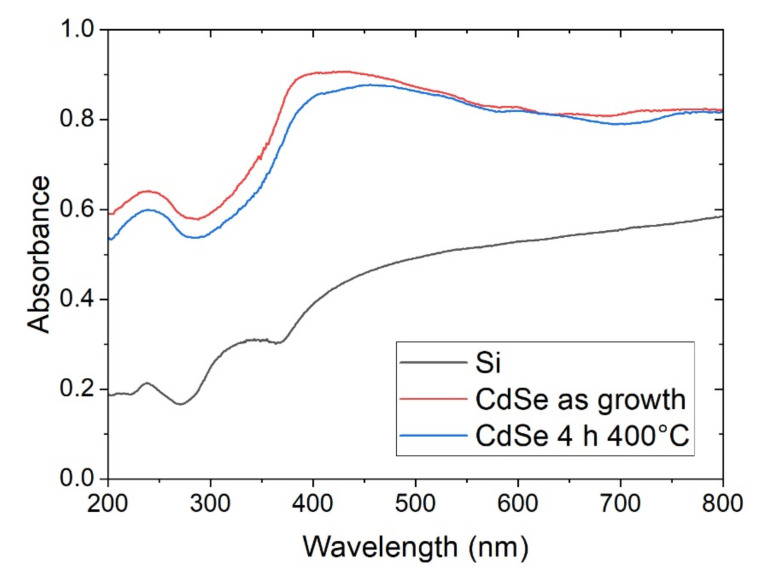
Absorbance spectra of the silicon substrate (black) and the sample prepared at −0.75 V: as growth (red) and after the 4 h annealing performed at 400 °C (blue).

The absorption spectra of the silicon substrate and of the CdSe were recorded before and after the heat treatment (Figure 6). We observed that the deposit has a much higher absorbance than the substrate. Instead, the annealing leads to a slight decrease in absorption. The variations in absorbance seem to confirm the results obtained with the AFM and XRD measurements, i.e., the annealing process leads only to a variation of surface morphology and therefore of scattering.

**Figure 7:**
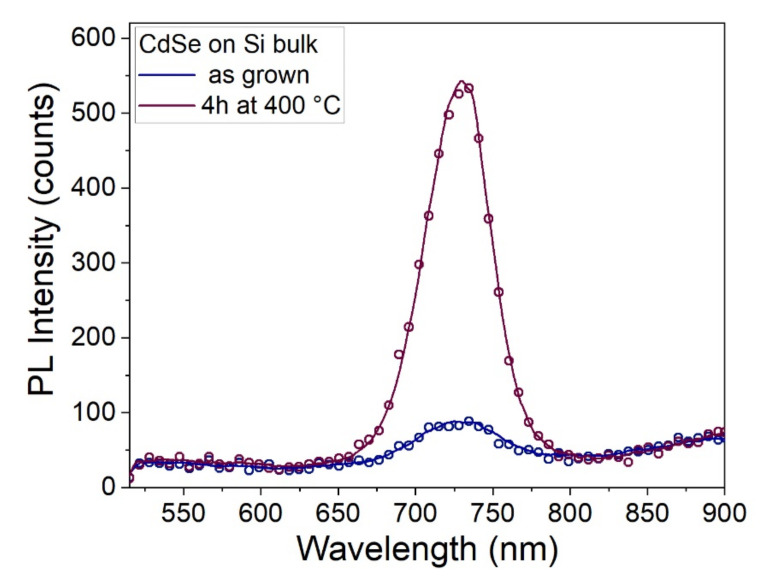
Photoluminescence spectra of the sample prepared at −0.75 V before (blue) and after (claret violet) the annealing at 400 °C for 4 h, using an excitation laser with a wavelength of 476 nm.

The photoluminescence spectrum of a sample prepared with a potential of −0.75 V was measured before and after the annealing at 400 °C for 4 h. The sample was excited with a 476 nm laser and the emission spectrum was recorded (more details in the experimental methods). After the annealing, the characteristic line shape of the emission peak of CdSe at 725 nm [53] remained unchanged but its intensity grew considerably by almost an order of magnitude in relation to the increase in crystallinity (Figure 7).

## 4. Conclusions

In this work, the optimal conditions for the deposition of a CdSe film on n-Si were achieved using a potential of −0.75 V and a solution of Na_2_SeO_3_ and 3CdSO_4_∙8H_2_O in sulfuric acid electrolyte. At an overpotential of lower than −0.70 V, an excess of Se was detected with RBS analysis, while at an overpotential of greater than −0.80, an excess of Cd was evaluated from electrochemical stripping voltammetry and SEM images. The crystallinity and photoemission of the samples were evaluated and, even if the electrodeposition provides a crystalline deposit, we found that after annealing at 400 °C under a nitrogen atmosphere, the PL increased by almost an order of magnitude. The electrochemical deposition is an easy and cost-effective preparation method and the results obtained in this study suggest that it could also be applied to the silicon-manufacturing processes for the realization of optoelectronic devices.

## Figures and Tables

**Table 1:** Atomic density and thickness obtained from RBS analysis of the films prepared at different potentials.

V Dep	Cd	Se	%CdSe	Thickness	
	× 10^16^ atm ∗ cm^−2^			CdSe (nm)	ε%
−0.60	1.78	2.86	76.7%	9.7	44.8%
−0.65	0.70	1.92	53.4%	3.8	17.6%
−0.70	3.10	3.09	99.8%	16.9	77.7%
−0.75	2.57	2.55	99.6%	13.9	64.1%
−0.80	2.07	2.10	99.3%	11.3	52.1%
−0.85	1.73	1.75	99.4%	9.4	43.5%
−0.90	1.17	1.37	92.1%	6.4	29.4%
−0.95	1.69	1.68	99.7%	9.2	42.3%

## Data Availability

Data are contained within the article.
